# Case Report: Novel rare *ZMPSTE24* variation in a Han Chinese family with early onset mandibuloacral dysplasia type B lipodystrophy

**DOI:** 10.3389/fendo.2026.1941254

**Published:** 2026-09-21

**Authors:** Shanshan Xue, Wujuan Shi, Na Zhang, Lihong Hao

**Affiliations:** 1 Department of Neonatology, Tianjin Children’s Hospital, Tianjin, China; 2 Department of Neonatology, Children’s Hospital, Tianjin University, Tianjin, China

**Keywords:** gene variations, lipodystrophy, MADB, mandibular hypoplasia, ZMPSTE24

## Abstract

**Background:**

Mandibuloacral dysplasia type B (MADB) is a rare autosomal recessive disorder characterized by heterogeneous manifestations, which is an extremely rare disorder with only a limited number of reported cases. Mutations in *ZMPSTE24* are associated with MADB.

**Methods and results:**

We reported a newborn who presented with core MADB manifestations (lipodystrophy, mandibular hypoplasia, joint contractures, shortened phalanges, and hypoplastic nails). We performed whole-genome sequencing and identified a rare novel a compound heterozygous *ZMPSTE24* variant [GRCh37] del(1) (p34.2) NC_000001.10:g.40747005_41013142del and g.40737681C > T. To date, only 12 pediatric MADB cases have been reported overall. Affected individuals typically exhibit heterogeneous manifestations, including skeletal abnormalities (mandibular and clavicular hypoplasia, acroosteolysis, delayed closure of cranial sutures, and joint contractures), cutaneous atrophy, lipodystrophy, and mottled cutaneous pigmentation.

**Conclusions:**

This study expands the genotypic and clinical phenotypic spectrum of *ZMPSTE24*-related MADB and reinforces the utility of whole-genome sequencing in early diagnosis and genetic counseling.

## Introduction

Mandibuloacral dysplasia type B (MADB) is a rare autosomal recessive disorder characterized by heterogeneous manifestations, including skeletal abnormalities (mandibular and clavicular hypoplasia, acroosteolysis, delayed closure of cranial sutures, and joint contractures), cutaneous atrophy, lipodystrophy, brittle hair and mottled cutaneous pigmentation. Mutations in *ZMPSTE24* are associated with MADB. MADB shares a genetic etiology with lethal neonatal restrictive dermopathy (RD) syndrome but differs by more generalized subcutaneous fat loss in the face, trunk, and extremities (1). However, MADB remains a rare condition worldwide, particularly in the neonatal period with early-onset presentation. In this study, we describe a newborn with a novel compound heterozygous pathogenic variant in the *ZMPSTE24* gene. High-throughput whole-genome sequencing (WGS) identified two distinct variants, including a large genomic deletion [GRCh37; NC_000001.10: g.40747005_41013142del(1p34.2)] and a missense variant (NC_000001.10: g.40737681C>T).

## Case presentation

The patient was the third child of healthy, non-consanguineous Han Chinese parents. He was delivered via cesarean section at 35 weeks of gestation due to placenta accreta, with no history of abnormal deliveries except for placenta previa and accreta. At birth, “generalized cutaneous edema (sparing the face and genitalia)” was noted, and the newborn was admitted to our hospital at 22 days of age because of “progressively worsened generalized cutaneous edema” in 2019. His parents and two elder sisters were healthy. On admission, his weight was 3080 g (birth weight: 2400 g). Physical examination revealed “generalized sclerodermoid skin with non-pitting edema”, cutaneous atrophy, and mottled pigmentation. Additional findings included short stature (47cm), short neck, large anterior fontanel (3.0 cm × 3.0 cm). Distinctive facial dysmorphisms comprised low-set ears, high-arched palate, micrognathia and retrognathia. He also exhibited a narrow barrel-shaped thorax, rounded shoulders, shortened phalanges, hypoplastic nails, and generalized joint contractures with limited mobility of all joints, including the fingers (**Figures 1A–D**). Primitive reflexes were absent. Furthermore, a prominent angioma measuring 3.0 cm × 3.5 cm was noted on the hip, along with enlarged breast nodules (**Figures 1E, F**). Written informed consent for genetic testing and publication of clinical information was obtained from the parents in accordance with the Declaration of Helsinki. Laboratory investigations confirmed anemia (hemoglobin: 104 g/L), hyperlactacidemia (6.24 mmol/L), and mild ketogenic bicarboxylic aciduria. No obvious abnormalities were detected in the patient’s blood glucose levels, hepatic and renal function, myocardial enzymes, thyroid function, or serum cholesterol concentrations. Ultrasonography revealed left renal pelvis dilation and patent foramen ovale. Electroencephalography indicated mild cerebral maturation delay, whereas head MRI showed widened extracerebral spaces and 3 mm pituitary gland height. Nerve conduction studies and electromyography of both the lower limbs were unremarkable. Skin ultrasonography confirmed reduced dermal and subcutaneous fat thickness in the lesional regions compared to normal skin (**Supplementaryay Figures 1A–C**). Skin CT revealed epidermal thinning and flattening in the lesional areas, increased dermal fibrous tissue refractive density, superficial dermal inflammatory cell infiltration, and sparse pericapillary structures with an intact basal cell layer (**Supplementaryay Figure 1D**). A skin biopsy showed no significant abnormalities (**Supplementaryay Figure 1E**). Cow’s milk protein is recognized as a leading cause of food allergy in infants, the patients was administered with extensively hydrolyzed protein formula for 5 days. However, the clinical response remained unsatisfactory. The neonate presented with “generalized cutaneous edema”, feeding intolerance, and poor weight gain. He was hospitalized and designated as patient 2. After performing the WGS, his parents requested discharge against medical advice. Radiographic imaging of the hands and feet was not obtained. WGS identified a novel compound heterozygous pathogenic variant in the *ZMPSTE24* gene, including a large genomic deletion [GRCh37; NC_000001.10: g.40747005_41013142del(1p34.2)] and a missense variant (NC_000001.10: g.40737681C>T) (**Figure 2A**). According to the American College of Medical Genetics and Genomics variant classification criteria, both identified variants were categorized as pathogenic. Specifically, the g.40737681C>T missense variant and the NC_000001.10:g.40747005_41013142del large genomic deletion fulfilled the same pathogenic evidence profile, including PS1 (Strong), PM3 (Moderate), PM2 (Supporting), and PP4 (Supporting) criteria. The father was heterozygous for the g.40737681C>T missense variant, whereas no pathogenic variant was detected in the mother (**Figures 2B, C**). This variant was not documented in the Genome Aggregation Database or the TOPMed/BRAVO datasets. Considering these findings, the diagnosis of MADB was established. After discharge,the patient suffered from feeding difficulties, poor weight gain, lipodystrophy and short stature. At 6 months of age, he was unable to tolerate complementary feeding, with persistent feeding intolerance and inadequate weight gain. By 1 year of age, he could neither stand unsupported nor develop verbal communication; additional findings included a retruded tongue base, severe short stature (length 50 cm, weight 4.1 kg), and delayed closure of the cranial sutures. At his most recent follow-up at 3.5 years of age (length 61 cm, weight 6.23 kg), formal development assessment confirmed his cognitive function equivalent to that of an 8-month-old infant, consistent with severe neurodevelopmental impairment. Regrettably, the patient’s family declined further diagnostic evaluation and therapeutic interventions, and the patient was subsequently lost to follow-up.

**Figure 1:**
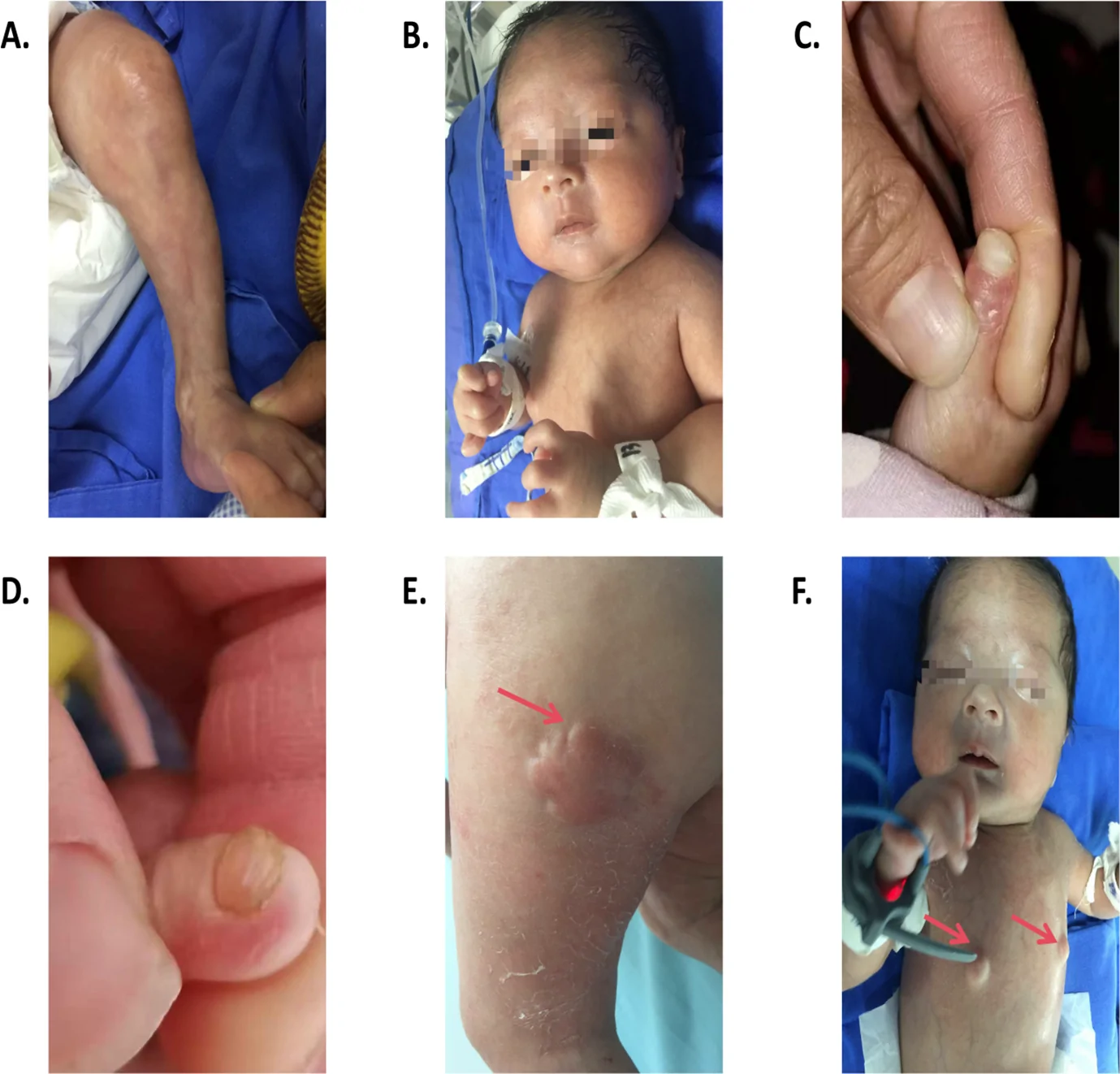
Clinical manifestations of the patient. **(A)** Markedly thin epidermis with tense skin, mimicking edema, and contracture of the knee joint. **(B)** Narrow barrel-shaped chest, rounded shoulders, shortened phalanges, and non-extensible fingers. **(C, D)** Hypoplastic nails in the proband’s thumb and forefinger. **(E)** A prominent angioma (3.0 cm × 3.5 cm) on the hip and enlarged breast nodules in the patient **(F)**.

**Figure 2:**
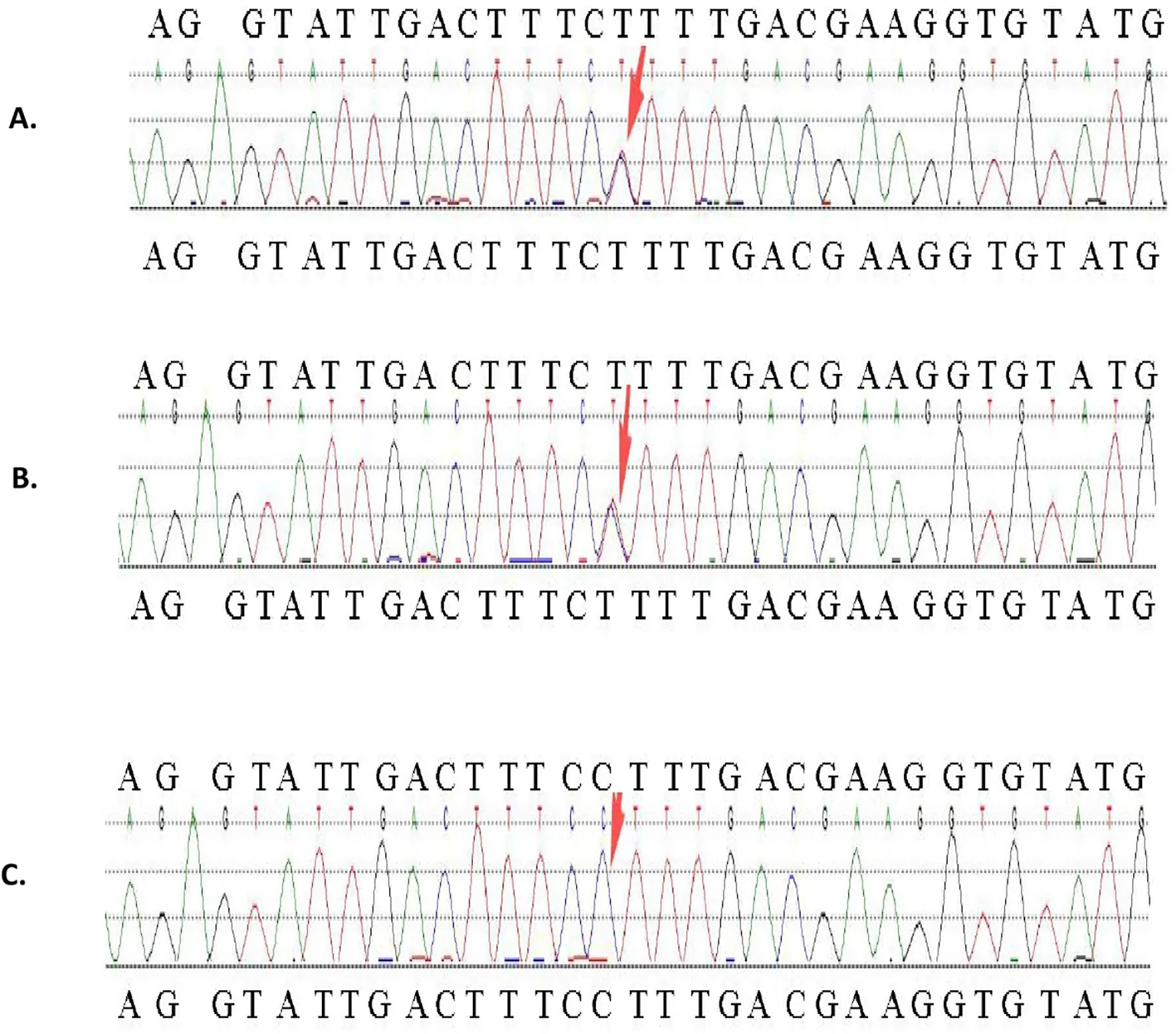
The genome sequencing of the patient and her parents. Genome sequencing demonstrated that there was a heterzygous variants (c.743C>T, p.Pro248Leu) and (c.793-1485del, p.Val265_Ser495del) of *ZMPSTE24* gene in the patient **(A)**, the mutation (c.743C>T, p.Pro248Leu) in his father **(B)**, and wide type in his mother **(C)**.

## Discussion and conclusions

MADB represents an extremely rare inborn error of metabolism, with only a limited number of neonatal cases documented in the literature. To date, 30 cases of MADB have been reported worldwide, all of which range from neonates to 41 years of age, only 12 pediatric MADB cases have been reported overall (1–11). The genotypic and clinical phenotypic data of pediatric cases with *ZMPSTE24* variants are summarized in **Table 1**. A review of pediatric MADB cases revealed that the majority of patients exhibited characteristic features, including short stature (11/11), clavicular hypoplasia (12/13), mandibular hypoplasia (13/13), delayed closure of cranial sutures (10/10), joint contractures (11/11), shortened phalanges (13/13), hypoplastic nails (11/11), acro-osteolysis of the distal (13/13), generalized lipodystrophic features (13/13). Moreover, MADB pediatric cases presented high palate (7/9), pinched nose (12/13), proptosis (7/12), and posteriorly rotated ears (7/10).

**Table 1:** Summary of variants of *ZMPSTE24* in individuals with MADB.

Patient	P1	P2	P3	P4	P5	P6	P7	P8	P9	P10	P11	P12	P13
**Origin**	Suriname	China	Pakistn	Chile	American	France	Japan		Portuguese	New Zealand	European		UK
**Consanguinity**	No	No	Yes	No	No	No	No		No	No	No		No
Clinical features													
Skeletal features													
Short stature	**++**	**+++**	N/A	**+**	**+**	**++**	**++**	**++**	**+++**	**+**	**+**	**+**	N/A
Clavicular hypoplasia	**++**	±	**+**	**++**	**++**	**-**	**+**	**+**	**+**,small	**+**,small	**+**, small	**+**	**+**, small
Mandibular hypoplasia	**+**	**+++**	**++**	**++**	**++**	**++**	**+**	**+**	**+**	**+**	**+**	**+**	**+**
Delayed closure of cranial sutures	**+**	N/A	N/A	**++**	**+**	**+**	**+**	**+**	**+**	N/A	**++**	**+**	**+**
Joint contractures	**+**	**++**	**++**	N/A	**++**	N/A	**+**	**+**	**+++**	**++**	**+**	**+**	**+**
Shortened phalanges	**++**	**++**	**+**	**++**	**++**	**++**	**+**	**+**	**+**	**++**	**++**	**+**	**+**
Hypoplastic nails	**+**	**++**	**+**	±	**++**	**++**	**+**	**+**	**+**	**+**	N/A	N/A	**+**
Acro-osteolysis of the distal phalanges	**++**	N/A	**+**	**++**	**++**	**+**	**+**	**+**	**+**	**++**	**+**	**+**	**+**
Lipodystrophic features													
Generalized	**++**	**++**	**++**	**++**	**++**	**++**	**+**	**+**	**++**	**++**	**+**	**+**	**+**
Extremities	**++**	**+++**	**+++**	**+++**	**++**	**++**	**+**	**+**	**++**	**++**	**±**	**±**	**+**
Truncal lipoatrophy	**++**	**++**	**++**	**++**	**+**	**++**	**++**	**+**	**++**	**++**	**+**	**+**	**+**
Facial lipoatrophy	**+**	**-**	**-**	**-**	**-**	**-**	**+**	**+**	**+**	**+**	**+**	**+**	**+**
Facial features													
High palate	**+**	**+**	**++**	**-**,small mouth	**+**	N/A	**+**	**+**	N/A	N/A	**+**	N/A	**-**
Pinched nose	**+**	**+**	**-**	**+**	**+**	**+**	**+**	**+**	**+**	**+**	**+**	**+**	**+**
Proptosis	**+**	**-**	**-**	strabismus	**+**	**-**	**+**	**+**	**+**	N/A	**-**	**+**	**+**
Posteriorly rotated ears	**+**	+	**-**	**-**	N/A	**-**	N/A	N/A	**+**	**+**	**+**	**+**	**+**
Gaze palsy /ptosis	**-**	**-**	**-**	N/A	N/A	**+++**	N/A	N/A	N/A	N/A	N/A	N/A	**-**
Tooth abnormalities													
Dental crowding	**++**	N/A ,delay	**-**	**++**	**-**	N/A	**+**	N/A	N/A	N/A	**+**	**-**	**-**
Hypoplastic teeth	**+**	N/A	N/A	N/A	N/A	N/A	Delayed eruption	N/A	Delayed eruption	N/A	Delayed eruption	N/A	**-**
Hair abnormalities													
Brittle / sparse hair	**++**	**+**	**++**	**+**	**-**	**++**	**+**	N/A	**+**	**+**	**+**	**-**	**+**
Alopecia	**+**	**++**	N/A	**+**	**-**	N/A	**-**	N/A	**+**	**+**	**+**	**+**	**-**
Skin abnormalities													
Mottled pigmentation	**++**	±	N/A	**++**	**++**	**+**	**+**	**+**	N/A	N/A	**+**	**+**	**+**
Skin atrophy	**++**	**++**	**++**	**++**	**++**	**++**	**++**	**++**	**++**	**++**	**+**	**+**	**++**
Sclerodermic skin	**++**	**+++**	**++**	**+**	N/A	**++**	**++**	**+**	**++**	**+**	N/A	N/A	**+**
Calcified skin nodules	**++**	**-**	N/A	**++**	**+**	N/A	N/A	N/A	**+++**	**+**	N/A	N/A	**-**
Developmental delay	**-**	**++**	N/A	**++**	**++**	**+++,**learning difficulties	**+**	**+**	Motor delayed +++, cognition normal mal;	N/A	Motor delayed ++, cognition normal	–	**-**
Growth retardation	**+**	**++**	N/A	**++**	**-**	**+++**	**-**	±	**+++**	N/A	**+**	**-**	**-**
**Microcephaly**	**++**	**-**	N/A	**-**	N/A	**++**	**+**	**-**	**+++**	**+**	**+**	**+**	**-**
**Metabolic disorders**	High blood glucose, blood lipid not assessed	normal blood glucose and lipid	N/A	N/A	N/A	moderate proteinuria	High blood glucose and lipid		polycythemia, high blood glucose and lipid, poor breast development	N/A	–	–	Mild dyslipidaemia, Neonatal alloimmune thrombocytopenia
**Other manifestations**	Underdev-eloped occipital bone,	mild HL, narrow and barrel shaped thorax, rounded shaped shoulders	Thoraco -lumbar -kyphosis	narrow and barrel shaped thorax, rounded shaped shoulders,	facial asymmet-ry and leg contract-ures, large fontanel-les	Failure of ossification of the interparietal region of the occipital bone,.	K, narrow and drooping shoulders, bilateral flexion contractures of elbows, hips and knees	K	CHR, hypotonia, myopathy, K	neonatal tooth eruption, submetaphyseal -erosions, vertebral beaking, severe cortical osteoporosis and delayed fracture healing	narrow sloping shoulders	–	–
**Prenatal findings**	N/A	Preterm, placenta previa and placenta accreta	N/A	Preterm, prenatal exposure to drugs	Preterm,	Preterm, generalized cutaneous edema sparing face and genitalia	IUGR		Preterm, hydramnios	Preterm	Preterm		Preterm
**cDNA**	c.1196A > G	c.743C>T, c.793-1485del	c.28_29insA	c.1085dup, c.794A>G	c.794A>G, c.1085T>A	c.1196A>G	c.121C>T, c.743C>T		c.281T>C	c.794A>G, c.207-208delCT	c.743C>T, c.1349G>A,		c.1085_1086insT, c.794A>G,
**Status**	Hom	Compound Het	Hom	Het	Het	Hom	Compound Het		Hom	Compound Het	Compound Het		Compound Het
**Protein change**	p.Y399C	p.P248L, p.V265_S495del	p.L10Y fs*37	p.L362F fs*19, p.D265S	p.N265S, p.L362fs*19	p.Y399C	p.P248L, p.Q41fs		p.L94P	p.D266S, p.Y70fs	p.P248L, p.W450fs		p.L362fs*19, p.N265S
**Reference (PMID)**	31856865	Novel	37270786	30919593	25629449	27410998	18435794		21267004	20550970	20814950		15937076

P, patient; M, male; F, female; -, absent; ±, very mild; +, mild; ++, moderate; +++, severe; N/A, not available; Het, heterozygosity; Hom, homozygosity, CHR, chronic renal failure; K, kyphoscoliosis; HL, hearing loss.

Herein, we report one of the youngest neonatal cases of MADB described to date, characterized by early onset and diagnosis. This case was caused by two novel compound heterozygous *ZMPSTE24* variant as a large genomic deletion [GRCh37; NC_000001.10: g.40747005_41013142del(1p34.2)] and a missense variant (NC_000001.10: g.40737681C>T), which clinically mimicked neonatal food allergy. Moreover, the patient presented with feeding intolerance, poor weight gain and neuromotor developmental delay. The patient was admitted primarily with a clinical impression of “progressive generalized cutaneous edema edema”; however, this finding likely did not represent true edema, but instead reflected thinning of the epidermis and tense skin. The proband presented with mottled atrophic skin, generalized lipodystrophy, and skeletal manifestations including growth retardation, mandibular and clavicular hypoplasia, and shortened phalanges. This clinical picture was further complicated by rare features (prominent angioma and enlarged breast nodules) that have been poorly documented in prior published reports. Additionally, the patient had short stature but lacked the evidence of acroosteolysis, which is a key skeletal feature of typical MADB.

There are some disorders that should be differentiated from neonatal MADB, particularly RD, mandibuloacral dysplasia type A (MADA), and other laminopathies. RD is a rare, lethal neonatal genodermatosis caused by mutations in *LMNA* or *ZMPSTE24*, which is characterized by taut, translucent skin, fixed arthrogryposis, facial dysmorphism, and early neonatal death. MADA lipodystrophy is a rare autosomal recessive disorder characterized by craniofacial dysmorphism, type A lipodystrophy, clavicular dysplasia, and acroostelolysis, which is caused by homozygous or compound heterozygous missense mutations in *LMNA* gene. Laminopathies are tissue-specific or systemic disorders caused by variants in *LMNA* gene (primary laminopathies) or in other genes encoding proteins which are playing some role in prelamin A maturation or in lamin A/C function (secondary laminopathies). Those disorders in which adipose tissue is affected are called laminopathic lipodystrophies and include type 2 familial partial lipodystrophy and certain premature aging syndromes. Therefore, the proband exhibited core clinical manifestations of MADB. Whole-genome sequencing and skin-related examinations were performed to confirm the final diagnosis of MADB.

*ZMPSTE24* encodes a nuclear membrane-embedded zinc metalloproteinase, also known as farnesylated protein-converting enzyme 1, which cleaves the carboxy-terminal residues of farnesylated prelamin A in two steps to form mature lamin A (12). MADB severity correlates with residual ZMPSTE24 enzymatic activity and the accumulation of unprocessed prelamin A. Loss of *ZMPSTE24* in articular cartilage could exacerbate the occurrence and development of osteoarthritis (13). A complete loss of function in *ZMPSTE24* was lethal in the neonatal period, whereas compound heterozygous mutations including one PTC and one missense mutation were associated with type B mandibuloacral dysplasia (8).

This case highlights that when neonates present with lipodystrophy, short stature, and skeletal anomalies - including micrognathia, hypoplastic clavicles, joint contractures, shortened phalanges, hypoplastic nails, and distinctive facial features, clinicians should pay close attention to differentiating MADB from other mimicking disorders to avoid misdiagnosis or underdiagnosis. To the best of our knowledge, this is the first report of MADB caused by compound heterozygous variants in *ZMPSTE24* [GRCh37; NC_000001.10: g.40747005_41013142del(1p34.2)] and a missense variant (NC_000001.10: g.40737681C>T). Given the father’s clinically unaffected status, we hypothesize that the missense variant is paternally inherited, whereas the large-scale genomic deletion arose *de novo* in the proband. The deleted genomic region involved 8 genes, including *ZMPSTE24*, *COL9A2*, *RPL21P20*, *SMAP2*, *ZFP69B* - *ZFP69*, *EXO5*, *ZFP684*. Based on the annotated biological roles of these deleted genes, *COL9A2* may be involved with the proband’s characteristic clinical presentations. This case expands the clinical and molecular spectrum of MADB and provides valuable insights for genetic counseling of affected families. Further observational and functional experiments are warranted to elucidate how the 266.137 kb deletion contributes to the development of survivable MADB rather than lethal RD. Early perinatal recognition of the distinctive clinical and cutaneous histological features, combined with molecular confirmation, facilitates timely genetic counseling (12).

This study has several limitations. First, the findings are based on a single case, which limits the generalizability of the results. Second, functional studies were not performed to confirm the pathogenic effects of the identified *ZMPSTE24* variants. Although bioinformatic analyses and population databases indicate that these variants are extremely rare, further experimental validation is needed to determine their precise molecular mechanisms. Third, the short follow-up period restricted the evaluation of the patient’s long-term clinical progression and outcomes. Owing to the patient’s young age, dual-energy X-ray absorptiometry, body composition analysis and metabolic work-up for insulin resistance were not performed. Future studies involving additional cases and functional investigations are required to better understand the genotype-phenotype relationship and the pathogenic mechanisms underlying MADB.

This study contributes to the existing body of evidence on *ZMPSTE24*-related clinical phenotypes and aids in optimizing prognostic assessment and therapeutic strategies for affected individuals.

## Data Availability

The raw data supporting the conclusions of this article will be made available by the authors, without undue reservation.

## Supplementary material

Supplementary Figure 1 The skin tissue structure in lesional regions and normal skin. Skin ultrasonography confirmed reduced thickness of the dermis and subcutaneous fat in lesional regions **(A, B)** compared to normal skin **(C)**. Skin CT revealed that thinning and flattening of the epidermis in lesional areas, increased refractive density of dermal fibrous tissue, superficial dermal inflammatory cell infiltration, and sparse pericapillary structures, with an intact basal cell layer **(D)**. Skin biopsy showed no significant abnormalities **(E)**.
